# Editorial: Redox Homeostasis and Cancer

**DOI:** 10.1155/2020/5487381

**Published:** 2020-12-18

**Authors:** Mithun Sinha, Adil Mardinoglu, Jayeeta Ghose, Kanhaiya Singh

**Affiliations:** ^1^Indiana Center for Regenerative Medicine and Engineering, Indiana University School of Medicine, Indianapolis, Indiana 46202, USA; ^2^Centre for Host-Microbiome Interactions, Department of Dentistry, Oral & Craniofacial Sciences, King's College London, London SE1 9RT, UK; ^3^Science for Life Laboratory, KTH Royal Institute of Technology, Stockholm 17121, Sweden; ^4^Comprehensive Cancer Center, Department of Radiation Oncology, The Ohio State University Wexner Medical Center, Columbus, Ohio 43210, USA

Under physiological conditions, a balance between oxidants and antioxidants exists. Reactive oxygen species (ROS) are continuously generated by aerobic cells and eliminated through scavenging systems to maintain redox homeostasis. Disruption of redox homeostasis results in oxidative stress and altered ROS signaling. Higher ROS levels can lead to DNA mutation and genomic instability which can play causal role in cancer development and progression. These mutations coupled with distorted redox signaling pathways orchestrate pathologic events inside cancer cells, resulting in resistance to stress and death signals, aberrant proliferation, and inefficient repair mechanisms.

Cancer cells are energy intensive, owing to their high rate of proliferation. However, due to impaired TCA cycle and poor blood perfusion, cancer cells switch towards glycolytic pathway for energy generation termed as “Warburg effect.” Such pathways lead to a higher oxidative environment. The oxidative environment is also enhanced by tumor-infiltrating macrophages and neutrophils. Thus, cancer cells are used to a high ROS environment. This redox imbalance allows for protumorigenic cell signaling. In this issue, we explore the relation between ROS and cancer. The issue comprises of twenty original articles and comprehensive reviews.

Reactive oxygen species (ROS) mediates cisplatin-induced cytotoxicity in tumor cells. However, when cisplatin-induced ROS do not reach cytotoxic levels, cancer cells may develop chemoresistance. Di Vito et al. reported the association of ferritin heavy subunit (FHC)-ROS axis with cisplatin resistance in ovarian cancer cells. Thus, implying that inhibition of FHC might be a potential approach for restoring cisplatin sensitivity of resistant ovarian cancer cells. The authors investigated whether the modulation of H-Ferritin might affect cisplatin-induced cytotoxicity in ovarian cancer cells. H-Ferritin knockdown strengthened cisplatin-mediated ROS increase and significantly restored sensitivity resistant ovarian cancer cells.

Studies by Zhao et al. in osteosarcoma cells found that metformin (drug for type 2 diabetes) suppressed the self-renewal ability of osteosarcoma stem cells (OSCs) and induced G0/G1 phase arrest by blocking the activity of cyclin-dependent kinases. Metformin triggered apoptosis in these cells, which promoted cell death *via* a ROS-dependent mitochondria-mediated pathway. Hämäläinen et al. investigated the expression of redox regulator nuclear factor erythroid-2-related factor (NRF)1 and NRF2 in skin lesions like naevi and melanoma from 172 patients. NRF1 and NRF2 are transcription factors essential for maintaining redox homeostasis and coordinating cellular stress responses. The study found the association of redox microRNAs (miRs) (miR-144, miR-212, and miR-510) with aggressive melanoma feature. This study opens up avenues to test these redox miRs as possible prognostic value in larger cohorts. The research article by Pires et al. described label-free mass spectrometry- (MS) based proteomics of breast cancer (BC) plasma to investigate the differences between circulating proteins between chemoresponsive and chemoresistant luminal A breast cancer. Protein-protein interaction networks were created utilizing using *in silico* tools. The study reports interesting findings on differences in inflammatory, complement system, and oxidative stress pathways in both BC phenotypes which holds potential clinical implications. Moreira et al. presented their findings about the antitumor effect of celastrol, a natural pentacyclic triterpenoid, in colon cancer cells with acquired resistant to cytotoxic drugs. Yang et al. have evaluated the anticancer and anti-invasive properties of alpha-lipoic acid (ALA) in gastric cancer cells. ALA is a naturally occurring thiol antioxidant which is known to exhibit antiproliferative and cytotoxic effects on several cancers. The study found that the Mucin 4 (MUC4) gene was strongly expressed in human gastric cancer tissues. ALA administration reduced the proliferation and invasion of human gastric cancer cells by suppressing MUC4 expression. Helfinger et al. reported expression of Nox4 in macrophages and the role played by them in determining the polarization and the phenotype of macrophages. In the report by Acheva et al., authors showed epithelial to mesenchymal transition (EMT) in lung cancer cells postexposure to oxidative stress of gamma radiation. The authors conclude that induction of EMT in bronchial epithelial cells by radiation requires more than single acute exposure to gamma radiation and that the presence of stromal component might enhance the effect through free radical production and accumulation.

Gao et al. investigated the association of nitric oxide metabolites and lung cancer incidence through a matched case-control study based on the German ESTHER cohort. The study deduced that subjects with high urinary nitrite/nitrate concentrations had an increased risk of lung cancer. Butturini et al. highlighted plant-derived Sesquiterpene as a potential for cancer therapeutics. The authors exhibited that the compound downregulated STAT3 signaling leading to an antitumor effect and correlated the anti-STAT3 activity with their ability to decrease GSH levels in cancer cells. These properties make them lead compounds for the development of a new therapeutic strategy for cancer treatment.

In addition to these original works, Kim et al. listed a comprehensive report on the ROS-inducing strategy in anticancer therapy. The review by Xian et al. summarized the role of ROS in cutaneous carcinogenesis and skin cancer progression. Akanji et al. provides a comprehensive account of hypoxia-inducible factors (HIFs). In the review by Lv et al., the authors discussed recent understanding in the involvement of Glutathione (GSH) in cell death pathways such as apoptosis, necroptosis, ferroptosis, and autophagy. Goh et al. performed online literature search to identify studies reporting metabolic biomarkers of aerodigestive squamous cell carcinomas (ASCC). Akbari et al. have focused on the role of hydrogen sulfide in bladder, kidney, and prostate malignancies.

In the review by Tataranni et al., the authors have addressed the therapeutic potential of dichloroacetate (DCA) in cancer therapy. DCA is a 150 Dalton, water-soluble acid molecule, analog of acetic acid in which two of the three hydrogen atoms of the methyl group have been replaced by chlorine atoms. The authors have summarized recent reports suggesting the employment of DCA in cancer therapy, in combination with chemotherapy agents, radiotherapy, and other chemical or natural compounds showing anticancer properties. Paz et al. in their review have discussed the pharmacological effects and toxicogenetic impacts of omeprazole in context of cancer. This extensive report highlights that omeprazole therapy may induce genomic instability and increase the risk of certain types of cancer and hence advocates for taking adequate precautions, especially in long-term therapeutic strategies. The review by Fiocchetti et al. focused studies conducted on Neuroglobin (NGB), a globin primarily described in neurons as an oxidative stress sensor and cytoprotective factor against redox imbalance.

Although ROS sustain tumorigenesis and cancer progression, these can also be efficient therapeutic tools to fight cancer. Oxidative stress-based therapies like radiotherapy, chemotherapeutic agents, and photodynamic theory increase ROS levels in the tumor niche and take advantage of the cytotoxic face of ROS for killing tumor cells by a nonphysiologically sudden, localized, and intense oxidative burst. Clinical efficacy of anticancer therapies is often subdued by multidrug resistance (MDR). Redox therapy by using redox-active drugs or inhibitors of inducible antioxidant defense in tumor microenvironment has reported to be effective against MDR tumors. Further insight into such redox biology will enable precisely targeted manipulation of ROS for effective medical therapies against carcinomas.

